# Obstetrics

**Published:** 1895-06

**Authors:** L. M. Griffiths


### OBSTETRICS.

Injuries to the mother occurring during labour do not always receive the attention they deserve. Whilst it is no doubt true that many lacerations of the perineum heal spontaneously, and whilst it is not desirable that every tear, however slight, should be sewn up as a matter of routine, yet an injury which is likely to require operation later on should be sutured immediately after its occurrence.

Lacerations of the cervix no doubt usually escape recognition at the time they occur. Amongst the many distressing consequences attributed to them have been named: alarming hemorrhage, septicæmic infection, menorrhagia, metrorrhagia, leucorrhœa, uterine displacements, sterility, and various neuroses. Emmet's well-known operation was devised to remedy these disasters, but it has never been very popular in this country. Much attention has been directed to the condition by American surgeons, and as long ago as 1880, at the Cambridge meeting of the British Medical Association, Pallen of New York recommended the immediate closing-up of all lacerations of the

| | Condition of Patient at Time of Operation. | Bilateral or Unilateral Castration. | Result. |
|---|---|---|---|
| 14 | Very large prostate. | B | On the 10th and 11th days after castration, blood-stained urine passed spontaneously. On the 11th day the patient died with extreme dyspnœa. At the P.M. the heart was found fatty and the kidneys cirrhotic. The bladder contained a calculus. The prostate had begun to atrophy, as shown by the wrinkled mucous membrane lying over it. |
| 15 | Prostate immensely enlarged. | B | In two months prostate diminished one-half; no trouble with micturition. |
| 16 | Aggravated cystitis. Night much disturbed by micturition. | B | Prostate atrophied; urine became nearly clear; all difficulty in micturition disappeared. |
| 17 | Very much enlarged prostate and "usual cystic symptoms." | B | Cystitis nearly disappeared; holds water much longer, but has to use catheter occasionally. Too early for complete report. |
| 18 | "Almost constant reminder that he has to void urine." | — | March 29th patient writes: "But little pus in urine; entire subsidence of pain; urinates but about once in twenty-four hours, and does not have to use catheter." |
| 19 | Twelve ounces residual urine, mixed with pus and blood. Very large prostate. | B | A week after, dysuria less, but died of hemiplegia Jan. 19th, 1895. |
| 20 | — | B | Died on sixth day of acute mania. |
| 21 | Enlarged prostate. | B | Mania came on, and died on twelfth day. |
| 22 | — | B | Report thirty days later gives no improvement. |

CHARLES A. MORTON.

cervix.[1] But his practice did not find many followers, and in 1889 Dr. Boldt, before the American Gynecological Society, suggested a modification which, consisting of a somewhat deferred operation, he termed "intermediate trachelorrhaphy."

Quite recently the subject has been prominently before some of the societies in the States. Dr. William R. Pryor[2] considers that hemorrhage is the only condition justifying immediate repair of the torn cervix. He considers that laceration of the cervix is not a cause of subinvolution, and he doubts the possibility of septic infection taking place by way of such a tear. Dr. Pryor says that a critical examination of the frozen sections of *post-partum* cases fails to show the cervical lips torn to the extent described by the earnest advocates of trachelorrhaphy. He considers that to apply the procedure in all cases where even marked separation of the cervical lips appears to exist is to introduce into obstetrics one more interfering, meddlesome routine operation for which there is absolutely no reason, and to

[1] *Brit. M. J.*, 1880, vol. ii., pp. 371-2; 1881, vol. i., pp. 764, 801.
[2] *N. York M. J.*, vol. lxi., 1895, p. 71.

add more chance for infection where already too many are presented by the aggressiveness of modern obstetrics. In many cases immediate suture restores a uterus to its first distorted state, and that often when there is a special necessity for the opportunity of free drainage after labour.

On the other side we have the enthusiasm of Dr. A. Palmer Dudley,[1] the Professor of Gynecology in the New York Post-Graduate Medical School, who was led to pay special attention to the subject by Dr. Boldt's paper. Dr. Palmer Dudley, independently of Pallen and others, came to the conclusion that the right practice was to sew up all tears immediately after delivery. He thinks that some of those who in the pre-antiseptic days viewed the operation with disapproval would now be in favour of it. He lays much stress on the point on which few would differ from him; viz., that the operation is emphatically called for in cases of severe hemorrhage caused by the laceration. He refers to twenty-one cases, in which he has performed immediate repair. His ordinary practice is to administer a small quantity of chloroform during the third stage of labour. Whilst waiting for the expulsion of the placenta, and when the patient is, from the effect of the chloroform, in a condition nearly allied to natural sleep, he examines the perineum, urethra, and vestibule to see if any injury has occurred. As the placenta is delivered the patient is kept in the Sims position. After washing and disinfecting his hands, Dr. Dudley explores the cervix by sweeping one or two fingers round it. If he discovers any lacerations he tells the relatives of the woman and gets one of the family to continue a few drops of the chloroform. If there is no relative available for this, cocaine applications must be used. He then introduces a large Sims speculum and gives it into the charge of the nurse. If any shreds of membrane still cling to the cervix, they are, after the vagina has been syringed with a solution of perchloride of mercury (1 in 5000), gently removed, and the lips of the cervix are caught with small forceps and brought into apposition. A pledget of sterilised gauze or cotton saturated with the perchloride solution is then introduced beyond the point of rupture. With a large-eyed Emmet cervix needle, curved, trocar-pointed, as many catgut sutures as are necessary are introduced. The pledget is removed, the cervix and vagina are well cleaned with the antiseptic lotion. The sutures are then tied with enough traction upon them to insure coaptation of the parts as the cervix contracts. Dr. Dudley gives details of five of his operations.

An interesting discussion followed the reading of Dr. Dudley's paper. Dr. C. C. Barrows advocated immediate repair, not only for the reasons given by Dr. Dudley, but also because uterine contraction was thereby favoured. Dr. Charles Jewett said that all tears of considerable depth should be sewn up at once if the obstetrician was also an experienced

[1] *Am. J. Obst.*, vol. xxxi., 1895, p. 145.

gynæcologist. But it must not be forgotten that a tear which at the close of labour might seem deep enough to require suture might appear unimportant after involution. Dr. W. T. Lusk said that he had for years always made immediate repair where it was possible to get permission. As the objection to immediate perineorrhaphy had been overcome, so he thought it would be in the case of the torn cervix. Dr. R. A. Murray supposed that there would be a general agreement about the desirability of repairing the cervix just after labour, if there were hemorrhage. But in such a case he did not think the operation very simple. Without using a speculum he preferred to pull down the cervix, a proceeding which in itself stopped the bleeding from the laceration. Catgut sutures should then be used. He also thought that all considerable lacerations should be sewn up even if they were not accompanied by hemorrhage.

Of twenty-four cases of *post-partum* hemorrhage occuring in the Rotunda in the year 1892-3, six are described[1] as being due to lacerations of the cervix. In three of these the rents were closed by suture. The others were treated by plugging with iodoform gauze.

Emmet's operation has not met with very much favour in this country, where it is usually considered that the evil results of a torn cervix are much exaggerated, but it is well that obstetricians should always remember the possibility of a severe hemorrhage being due to this cause.

* * * *

In the report on obstetrics in June, 1893, I referred to some papers on the care of pregnant women. Dr. William B. Dewees has recently been devoting much attention to this question.[2] He says that we must use our power for the prevention as well as for the alleviation of the sufferings of pregnant women. The study of the diseases and difficulties encountered with a state of pregnancy and parturition, though highly interesting and having at all times attracted the greatest attention of physicians, is, however, exceedingly difficult. This circumstance probably accounts for the fact that our obstetrical literature still continues to be full of errors. By consulting the best practical writers, missionaries, and travellers, the revelation comes that it is among the higher classes in the more civilised countries—Europe and North America—that women suffer the greater in both pregnancy and parturition; but that the women of the peasantry and the savage are comparatively exempt. We may therefore very naturally and wisely conclude that it is unnatural for civilised woman to suffer so universally as she does to-day ; and that civilisation is disobedience to the law of Nature, is the true cause of her present suffering during gestation and childbirth. The progress of obstetrics in the immediate future must be

[1] *Dublin J. M. Sc.*, vol. xcix., 1895, p. 11.
[2] *J. Am. M. Ass.*, vol. xxiii., 1894, p. 499 ; and *Internat. M. Mag.*, vol. iii., 1895, p. 794.

made through the knowledge that will be wrought out by the devotees of biology. Thus, we shall find our way, on positive ground, back through the morphology of organs, tissues, cells, and blood, to a clear comprehension of the origin of vital activity in protoplasm and the pabulum which sustains it. This is the only way open for us into the primitive arcana of Nature, if we would have the wisdom essential to intelligently inculcate that regimen which will successfully prevent the needless sufferings of pregnant and parturient women.

The general practitioner should be made to realise that the diseases peculiar to women during pregnancy and parturition are very largely preventible. It is within the power of the family physician very largely to prevent many of the diseases among the women entrusted to his care. When through him adequate instructions are given to married women, and the requisite attention is paid to growing girls, so that while at the age of puberty more vigorous bodies and more normal reproductive organs will be developed, the day will come when it will be no longer possible to define woman as "an animal with a constipated bowel and a pain in her side."

Dr. Dewees classifies as follows the obstetrician's paramount duties in reference to the women who come under his care:—(1) To discover if the patient be actually pregnant. (2) To determine positively if the impregnation be uterine or normal, as contradistinguished from tubal, abdominal, or abnormal pregnancy. (3) To carefully note the pregnant woman's history, including her age, primiparity or multiparity, environments, station in life, general condition of health, period of gestation; as well as her dress, food, drink, and habits of life. To make repeated examinations of the urine and ascertain the temperature, from the time pregnancy is established to the termination of gestation. (4) To make a physical examination for the purpose of accurately determining the diameters of the pelvic straits; the symmetry and size of the bony outlet; the integrity, condition, and position of the vagina, uterus, and other intra-pelvic viscera, and adjacent structures; the state of the abdominal muscles; the presence or absence of hernia, varicose veins, tumours, &c.; the shape, size, and condition of the breasts and nipples; the condition of the heart, lungs, mind, stomach, bowels, &c. (5) To observe the state of the fœtus, its strength and viability, as well as the implantation of the placenta.

* * * *

Bacteriology will no doubt have to play an increasing part in this prevention of some of the disasters to which pregnant and parturient women are liable, and antiseptic midwifery, although it has done something, has not nearly achieved the results of which it is capable. This is shown by the quantity of literature still urging its scientific adoption; and the little influence it has exercised in diminishing the mortality from puerperal fever in private practice has been emphatically pointed out by Dr.

Boxall[1] and others. Dr. W. P. Gamber declares[2] that he who keeps pace with the literature of medicine must acknowledge that the various forms of micro-organisms are the prime factors in the production of a large majority of the diseases in the human family, and some of them are certainly instrumental in the causation of puerperal fevers, although the pyogenic bacteria are widely distributed and are found on healthy mucous membrane, where they do no harm till some local lesion leads to septic infection. Of these the streptococcus pyogenes is the most important in reference to puerperal infection,[3] although the staphylococcus aureus has probably something to do with the milder cases of puerperal fever. The faithful practice of aseptic or antiseptic precautions consists (1) in preventing the introduction of germs, (2) in destroying the action of these germs if they have obtained admittance, and (3) in shutting up the doors by which their continued ingress would be gained. The first indication is best fulfilled by the perchloride of mercury method, of which we have heard so much, but this must be carried out thoroughly. It is matter of satisfaction to find that Dr. Gamber considers that a complete bathing of the lochial discharge from the genitals at least twice a day is of more importance than the vaginal douche. The second point mentioned by Dr. Gamber requires an active interference first by means of a vaginal douche once or twice in the twenty-four hours: if of carbolic acid or of creolin, the strength, he says, should be two per cent.; and if the perchloride is used, it should have the strength of one in 2,000. If these do not accomplish their purpose, the advisability of using the curette and intra-uterine douche should be considered. Dr. Cullingworth prefers using his fingers.[4] Dr. Gamber carries out the theory of his third heading by doing everything possible to maintain uterine contraction.

Immunised serum has found its way into obstetric practice. Puerperal septicæmia has been treated by injections of the serum of animals rendered immune to the streptococcus infection. The curative serum is prepared on the same general principles as the diphtheria antitoxin. At a meeting of the Société de Biologie on February 23rd, 1895, Roger reported a case of puerperal fever treated by Charrin and himself with streptococcus antitoxin. "Despite the gravity of the infection," he says, "this woman got well in forty-eight hours." At the March meeting he reported another case treated in the same way and with the same success. "What struck us particularly in both cases," he adds, "was the prompt amelioration of the general state, the sense of comfort experienced a few hours after the injections, and the speediness of the convalescence." [5]

* * * *

An admirable summary of the antiseptic methods adopted in

[1] *Antiseptics in Midwifery*, 1894. [2] *Med. & Surg. Reporter*, vol. lxxi., 1894, p. 97. [3] *A Manual of Bacteriology*, by George M. Sternberg, 1893, p. 279. [4] *Practitioner*, vol. liv., 1895, p. 315. [5] *Boston M. & S. J.*, vol. cxxxii., 1895, p. 444.

various midwifery clinics is given in the *Practitioner* for March, 1895. It closes by saying that to make antiseptic methods too complicated and too elaborate often ends in their being discarded altogether, with the result that the patient is exposed to grave risk, either of death or of serious and protracted illness. The nurse should be instructed that, as soon as labour-pains begin, she is to wash the patient's vulva with soap and water, and then thoroughly sponge it with cotton-wool soaked in an antiseptic solution. Before making an examination, the doctor should thoroughly cleanse his hands with hot water, soap, and a nail-brush, and immerse them for at least a minute in a similar antiseptic solution. The immersion should be repeated before every subsequent examination of the patient. It should never be forgotten that all the knowledge of the accoucheur goes for nothing if he be not imbued with the antiseptic method. After the labour is over, I am in the habit of ordering the introduction of a twenty-grain iodoform pessary into the vagina twice a day for the first three days and once a day afterwards for the next six days, and directing that there shall be no vaginal syringing.

* * * *

No obstetrician, unless he is a person of complete leisure, rejoices when he meets with a case of rigid os which maintains its provoking smallness for hour after hour; and although the administration of thirty grains of chloral in two doses at an interval of twenty minutes, as originally suggested by Dr. Playfair, will often induce it to mend its ways, it is well to have something else short of incision to fall back upon in case of failure. Dr. Joseph Farrar,[1] whilst recently attending a case of this sort in which various therapeutic resources were tried without good results, was about to incise the margin. For local anæsthesia he used a ten per cent. solution of cocaine, applying it by means of a linen rag, smearing it round and round on the end of the finger, and leaving it there for three or four minutes. When he introduced a finger as a guide to the scissors he found the previously rigid os widely dilated. Although he came to the conclusion that the cocaine had caused this rapid change, he did not report the case till he had another opportunity of trying the same treatment. This occurred soon afterwards in a case in which the os failed to dilate after labour had been going on for three days. Four minutes after the application of the cocaine the os yielded and became easily distensible.

In the discussion which followed Dr. Farrar's communication it was pointed out that the local use of cocaine in tedious labour was not new, but that it had not been hitherto attended with the beneficial results reported by Dr. Farrar.

L. M. Griffiths.

[1] *Tr. Obst. Soc. Lond.*, vol. xxxvi., 1895, p. 321.

